# Delving into the Antiurolithiatic Potential of *Tribulus terrestris* Extract Through –*In Vivo* Efficacy and Preclinical Safety Investigations in Wistar Rats

**DOI:** 10.1038/s41598-019-52398-w

**Published:** 2019-11-04

**Authors:** Jyoti Kaushik, Simran Tandon, Rishi Bhardwaj, Tanzeer Kaur, Surinder Kumar Singla, Jitender Kumar, Chanderdeep Tandon

**Affiliations:** 1 Amity Institute of Biotechnology, Amity University Uttar Pradesh, Noida, India; 2 Amity Institute of Molecular Medicine & Stem Cell Research, Amity University Uttar Pradesh, Noida, India; 3 Department of Biophysics, Panjab University, Chandigarh, India; 4 Department of Biochemistry, Panjab University, Chandigarh, India

**Keywords:** Drug safety, Urological manifestations

## Abstract

Modern treatment interventions for kidney stones are wrought with side-effects, hence the need for alternative therapies such as plant-based medicines. We have previously documented through *in vitro* studies that statistically optimized aqueous extract of *Tribulus terrestris* (*Zygophyllaceae* family) possesses antiurolithic and antioxidant potential. This provides strong scientific foundation to conduct *in vivo* efficacy and preclinical safety studies to corroborate and lend further proof to its ability to prevent and cure kidney stones. The preventive and curative urolithiatic efficacy in experimentally induced nephrolithiatic Wistar rats, along with preclinical toxicity was evaluated following oral administration of statistically optimized aqueous extract of *T*. *terrestris*. Treatment showed augmented renal function, restoration of normal renal architecture and increase in body weight. Microscopic analysis of urine revealed excretion of small sized urinary crystals, demonstrating that treatment potentially modulated the morphology of renal stones. Tissue enzymatic estimation affirmed the antioxidant efficacy of treatment with reduced free radical generation. Significant upregulation of p38MAPK at both the gene and protein level was noted in hyperoxaluric group and interestingly treatment reversed it. Acute oral toxicity study established the Median Lethal Dose (LD_50_) to be greater than 2000 mg/kg body weight (b.wt.) No observed adverse effect level (NOAEL) by repeated oral toxicity for 28 days at 750 mg/kg b.wt. was noted. This study lends scientific evidence to the safe, preventive and curative potential of statistically optimized aqueous extract of *T*. *terrestris* at a dose of 750 mg/kg b.wt. and suggests that the extract shows promise as a therapeutic antiurolithic agent.

## Introduction

Nephrolithiasis is one of the frequent and third most prevalent multifactorial disorders, affecting mankind globally. Moreover, the ensuing inflammation can lead to renal insufficiency which has been linked to disorders such as diabetes mellitus, obesity and hypertension^1^. Mineralized crystal deposition due to supersaturation of urine within the renal calyces and pelvis is responsible for kidney stone formation and contributes to renal dysfunction^2^. The complexity coupled with reoccurrence of kidney stones is a major concern and therefore warrants specific medical attention as there is an unmet need for efficacious therapies for the prevention of kidney stones.

Calcium oxalate crystals cause injury to renal cells and set into motion a series of events which lead to the generation of reactive oxygen species, which are then responsible for damage due to lipid peroxidation (LPO)^3^. Free radical mediated LPO damage to the renal cell membrane has been shown to be a key factor for not only the deposition of calcium oxalate crystals, but also their retention^4^. This has prompted the use of antioxidant herbal drugs and formulations as antilithiatic agents^5^ which have also been associated with lowered risk of adverse effects. *Tribulus terrestris *(*T*. *terrestris*), puncture vine from Zygophyllaceae family, possesses various pharmacological properties, which include antiurolithic, diuretic, anti-inflammatory and immunomodulatory *in vitro* and *in vivo* effects^6^. Our preliminary *in vitro* studies provided the scientific basis and important insights into the anticalcifying potential of the statistically optimized aqueous extract of *T*. *terrestris*^7^. However, to extrapolate the results obtained in the cell lines to the dynamic animal model, in-depth *in vivo* studies are necessary. This would throw light on the anticalcifying potential of the statistically optimized aqueous extract of *T*. *terrestris*.

## Materials and Methods

### Plant material and preparation of aqueous extract

The dried and matured fruits of *T*. *terrestris* were obtained from Natural Remedies Pvt.Ltd., Bangalore, India and voucher specimen is available with the company. Aqueous extract was prepared for oral dosing using methods described in our previous study^7^. Briefly, the dried and matured fruit of *T*. *terrestris* was ground into fine powder and the extraction was carried out at temperature of 23.5 °C for a period of 19.50 hours under constant stirring and solid to liquid ratio of 1 g /12 mL of the solvent (water). Following this, the extract was filtered, lyophilized and stored in airtight containers at −20 °C.

### Animals

Healthy male Wistar rats weighing between 150–200 g were obtained from Central Animal House of Panjab University, Chandigarh, India. They were maintained in propylene cages (47 cm × 34 cm × 18 cm) at 25 ± 1 °C and dark/light cycle of 12 hour, with food and water *ad libitum*. The male rats were divided with matched body weights into six groups of six animals each, which were then randomly selected to receive either the preventive (n = 36) or the curative treatment (n = 36). Experiments were conducted in accordance with accepted standard guidelines for the care and use of animals in scientific research and approved by the Institutional Animal Ethical Committee, Panjab University, Chandigarh(PU/IAEC/S/15/117). For the pre-clinical toxicity studies conducted at INTOX, Pune, India, a total of 58 rats of both sexes were used. The experimental design is discussed under the section on preclinical toxicity studies.

### Kidney stone induction and experimental design

**Figure 1:**
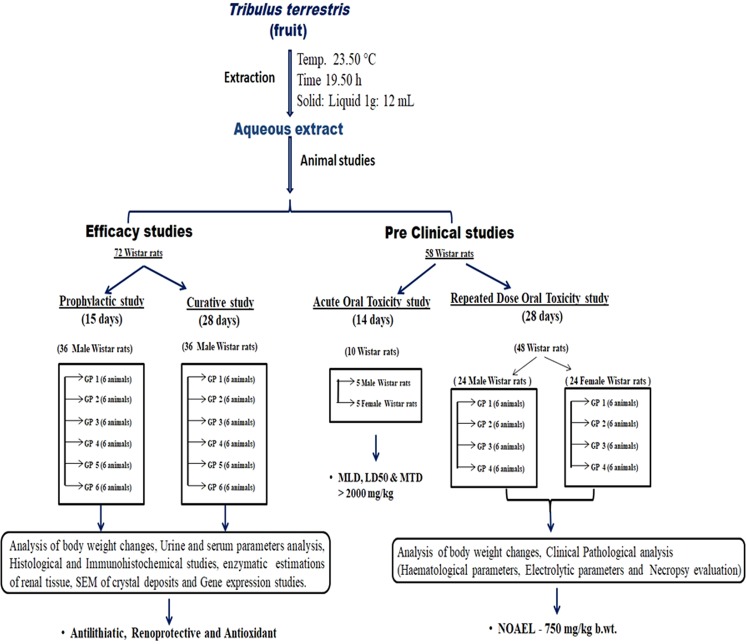
Flowchart depicting the Study Design.

A solution of 0.4% (v/v) Ethylene glycol (EG) and 1% Ammonium chloride (w/v) was used to induce hyperoxaluria in male Wistar rats^8^ for the formation of renal calculi. For *in vivo* studies, animals were divided into two major regimens according to the treatment period *i*.*e*. prophylactic regimen (PR, n = 36) 15 days and curative regimen (CR, n = 36) 28 days of treatment period. Each regimen was further subdivided into 6 groups having 6 animals each and represented as GP1 (control), GP2 (hyperoxaluric), GP3 (75 mg/kg *T*. *terrestris*), GP4 (225 mg/kg *T*. *terrestris*), GP5 (750 mg/kg *T*. *terrestris*) and GP6 (750 mg/kg cystone). The experimental design of the study is shown in Fig. 1.

### Dosage

Lyophilized powder of *T*. *terrestris* extract and standard drug (cystone) were re-suspended in drinking water and a single oral dose/per day was administrated to the animals of experimental groups. Cystone is a polyherbal formulation used for the management of kidney stones and is manufactured by The Himalaya Drug Company, Bangalore, India. Cystone contains various plant extracts such as *Didymocarpus pedicellate*, *Saxifraga ligulata* and *Gokshura* which have been shown to possess antilithic as well as diuretic properties. It has been reported previously that cystone at a dose of 750 mg/kg b.wt. per oris (p.o.) elicited protection against hyperoxaluria-induced oxidative stress and calcium oxalate crystal deposition. Therefore, we used this concentration for the cystone treated positive control group^9^.

### Prophylactic regimen (PR)

Rats in GP1 served as control and received regular feed and drinking water *ad libitum*. In GP2, hyperoxaluric condition was created by administrating 0.4% EG and 1% Ammonium chloride in drinking water for 15 days. Animals of GP3-GP5 received calculi inducing treatment (EG, 0.4% v/v + NH_4_Cl, 1% w/v) along with the administration of 75 mg/kg b.wt., 225 mg/kg b.wt., or 750 mg/kg b.wt. of aqueous extract of *T*. *terrestris* respectively. GP6 received 750 mg/kg b.wt. of cystone along with the calculi inducing treatment. The prophylactic regimen was undertaken to assess the potential of *T*. *terrestris* as a preventive agent for kidney stone formation.

### Curative regimen (CR)

GP1 was the control group and received regular feed and drinking water *ad libitum*. GP2-GP6 received calculi induction treatment (EG, 0.4% v/v + NH_4_Cl, 1% w/v) in drinking water for 15 days to hasten lithiasis, followed by 0.4% EG in drinking water from 16^th^–28^th^ day. Rats in GP2 served as hyperoxaluric group, in which the condition was maintained in the absence of any curative intervention. Animals of GP3-GP5 received doses of aqueous extract of *T*. *terrestris* at 75 mg/kg b.wt., 225 mg/kg b.wt., 750 mg/kg b.wt. respectively, while GP6 received cystone 750 mg/kg b.wt. along with 0.4% EG in drinking water from 16^th^–28^th^ day. Cystone treated groups served as positive control. The curative regimen was undertaken to assess *T*. *terrestris* as a potential curative agent.

### Experimental protocol

Body weight of all animals of the various groups of the regimens was recorded daily to keep check on their dietary intake and physical health. Urine sample was collected by keeping the rats overnight in metabolic cages fixed with urine collectors (15^th^ day from the rats in the prophylactic regimen and 28^th^ day from the rats in curative regimen). In the collected urine, 20% of sodium azide was added as an antimicrobial and preservative agent. Biochemical parameters of urine and serum samples were estimated by using commercially available diagnostics kits from Erba Diagnostics, Baddi, India. Urine samples were stored at 4 °C and spectrophotometric (Spectro UV-1800 Spectrophotometer, Shimadzu) determination of calcium (Cat. No. 120225) at wavelength 578 nm, magnesium (Cat. No. DB0938) at wavelength 520 nm, phosphorous (Cat. No. 120229) at wavelength 340 nm, uric acid (Cat. No. 120216) at wavelength 505 nm and alkaline phosphatase (ALP) (Cat. No. 120238) at wavelength 405 nm was performed. After urine collection, rats were anaesthetized with diethyl ether^9^ and blood was collected in centrifuge tubes from retro-orbital sinus under anaesthetic condition. For serum collection, blood was centrifuged at 10,000 rpm for 15 minutes and commercially available kits obtained from Erba Diagnostics, Baddi India, were used to spectrophotometrically measure the level of blood urea nitrogen (Cat. No. 120215) at wavelength 340 nm, creatinine (Cat. No. 120246) at wavelength 510 nm and uric acid (Cat. No. 120216) at wavelength 510 nm.

### Morphological evaluation of urinary crystals through microscopy

A drop of urine was spread uniformly on a clean glass slide, covered with cover slip and observed under Leica DM3000 light microscope^10^ at magnification 10X and 20X. Multiple fields were assessed for each sample.

### Histopathological and immunohistochemical analysis

Histopathological changes were analysed by haematoxylin and eosin staining^11^. Sections were observed under Nikon eclipse, Ti microscope at 20X and 40X and multiple fields were analyzed for all sections. Immunohistochemical staining was done by using p38MAPK antibody (sc-271120, Santa Cruz) at dilution 1:50 and Alexa flour 555 conjugated secondary antibody (Thermo Scientific). 4,6-diamidino 2-phenylindole -DAPI (5 μg/ml) was used as the counterstain. Laser confocal fluorescence microscope (Nikon Eclipse Ti-E) was used for imaging. Multiple fields were assessed from each section at 60X.

### Scanning electron microscopy (SEM) of renal crystals deposits

Kidney sections were de-paraffinised in xylene for 2 hours and downgraded through various grades of alcohol (90% to 45%) to water. The sections were exposed to beam of electrons for coating^12^. Each section was analyzed under scanning electron microscope (ZEISS EVO^®^ HD, Germany). Multiple fields were assessed between the range of 514X-1.04KX magnification, scale bar 20 µm and insert images were captured at higher magnification to analyse the morphology of stones, scale bar 2 µm.

### Enzymatic estimation of renal tissue

Renal tissue was excised, weighed and cut into small pieces and 10% (w/v) homogenate prepared in 0.1 M PBS buffer (pH 7.4) and lipid peroxidation^13^, reduced glutathione (GSH) and protein^14,15^ estimations carried out. The levels of catalase was assessed in the Post mitochondrial fraction^16^.

### Gene expression study

Total RNA was extracted from kidney tissue using TRI reagent. 2 μg of total RNA was reverse transcribed by verso cDNA synthesis kit (AB-1453/A, Thermo Scientific). Amplification of p38MAPK gene was performed by using PCR green mix (K1081, Thermo Scientific) for 35 cycles (94 °C for 30 s, 60 °C for 1 min, 72 °C for 30 s) using primers (sense 5′ CGAAATGACCGGCTACGTGG 3′, antisense 3′ CACTTCATCGTAGGTCAGGC 5′) in thermocycler. β-actin was amplified as an internal control with primers (sense 5′ GCTACAGCTTCACCACCACA3′, antisense 3′ ATCGTACTCCTGCTTGCTGA 5′). PCR products were electrophoresed on (1% w/v) agarose gel. Densitometrical measurement of band intensity was achieved by using Image J 1.48 software and values were normalized with those of β-actin.

### Preclinical toxicity studies

Preclinical toxicity study comprising of acute and repeated oral toxicity was conducted at INTOX PVT. LTD. Pune, Maharashtra, India. The studies were in compliance with the Principles of Good Laboratory Practice as set forth in Organization in Economic Co-operation and Development (OECD) 1998 and compliance Monitoring, Number 1, “OECD Principle on Good Laboratory Practice” ENV/MC/CHEM (98)17. Study was performed under the conditions recommended by the Committee for the Purpose of Control and Supervision of Experiments on Animals (CPCSEA) in the Guideline for Laboratory Animal Facility, India, (Under: Ministry of Fisheries, Animal Husbandry and Dairying, Department of Animal Husbandry and Dairying, Government of India).

### Acute oral toxicity study design

For acute oral toxicity study, aqueous, extract of *T*. *terrestris* was administered as a single oral gavage at limit dose of 2000 mg/kg b.wt. to a group of five male and five female rats (n = 10). Rats were observed for signs of toxicity and incidence of mortality and sacrificed after 14 days to evaluate gross/microscopic pathology.

### Repeated oral toxicity study design

Animals were divided into four groups with 6 rats in each group. Aqueous extract of *T*. *terrestris* was administered orally to 48 rats (24 female + 24 male) daily for the period of 28 days. Details of doses are shown in Table 1. Animals were examined daily for signs of toxicity and mortality. Further, rats were subjected to detailed clinical examination as per the OECD guidelines, in which any changes in fur, eyes, mucous membranes, occurrence of unusual secretions and excretions and alterations in autonomic activity such as lacrimation, piloerection, pupil size, respiration or gait were noted. This exercise was done both before initiation of the treatment and weekly thereafter during the treatment period by the veterinarian outside the home cage in a standard arena, usually at the same time. Body weights of all animals were recorded before initiation of treatment (day 0), once in a week (on days 7, 14, 21 and 28) and at necropsy (day 29). Food consumption was also recorded weekly. Blood and plasma samples of all animals were subjected to clinical haematology and clinical chemistry evaluations at the end of the treatment. Analysis of urine was performed in the last week of treatment. At the end of treatment, all animals were sacrificed for detailed necropsy. Organ weights (kidney, liver, heart, adrenals spleen, testis, uterus and brain) were also recorded for all animals and histopathological evaluation performed for each group.

**Table 1:** Details of dosing for repeated oral toxicity study.

Dose Group	Dose (mg/kg)	Dose	Frequency of treatment	Male rats	Female rats
GP1	0 (Vehicle Control)	0	Once daily	6	6
GP2	75 mg/kg	2	Once daily	6	6
GP3	225 mg/kg	4	Once daily	6	6
GP4	750 mg/kg	8	Once daily	6	6

### Statistical analysis

Data were expressed as mean ± standard deviation. Mean difference among different groups were analysed using analysis of variance (ANOVA). Homogeneity of data was checked by utilizing Bartlett’s test. In order to find out significant differences between groups, posthoc analysis utilizing Tukey test was performed. A p value < 0.05 was considered to be significant. The statistical software Graph Pad Prism version 6.01 was employed to perform statistical analysis.

## Results

### Changes in body weight, biochemical analysis of urine, serum and measurement of antioxidant status in renal tissue

Induction of renal stone formation led to a significant decrease in body weight in both the prophylactic and curative regimens when compared with untreated control rats (GP 1). The hyperoxaluric rats (GP 2) lost 72.50 g (loss of 38%) and 84.50 g (loss of 43%) of mean weight in the PR and CR rats, respectively. The biochemical analysis revealed that co-treatment with the aqueous extract of *T*. *terrestris* improved the overall metabolic activity of animals, which was seen by the dose dependent weight gain in each of the groups (Supplementary Tables 1 and 2). In the group treated with the highest dose of the extract *i*.*e* 750 mg/kg. b.wt. (GP5), the weight was comparable to the cystone treated rats (GP6) in both regimens, PR (Table 2) and CR (Table 3). Urine and serum analysis showed that upon treatment with aqueous extract of *T*. *terrestris*, the electrolyte balance was potentially restored compared to the hyperoxaluric rats (Tables 2 and 3). Levels of urinary calcium, uric acid, phosphorous and alkaline phosphatase were also reduced in the extract treated animals. However, magnesium levels increased in treated groups of both PR and CR rats compared to the hyperoxaluric GP2 rats. Elimination of nitrogenous waste was also observed from the kidney of experimental groups, as *T*. *terrestris* treatment led to a concomitant reduction in blood urea nitrogen, uric acid and creatinine in both PR (Table 2) and CR (Table 3) with respect to GP2. Our results revealed that treatment with the extract at dose of 750 mg/kg b.wt. showed maximum antilithiatic potency and was comparable to the cystone treated rats (GP6).

**Table 2:** Assessment of physiological parameters (body weight, urine biochemistry, serum biochemistry and enzymatic estimation) in rats on prophylactic regimen.

Parameter	GP 1	GP2	GP 3	GP 4	GP5	GP 6	P Value*
Body Weight (g)	189.83 (6.08)	117.33 (3.33) {−38%}	139.17 (7.03) {−27%}	160.17 (8.61) {−16%}	165.00 (5.83) {−13%}	168.83 (8.06) {−11%}	<0.0001
Urinary Calcium (mmol/L)	0.29 (0.07)	2.28 (0.28)	1.90 (0.19) [−17%]	1.72 (0.18) [−25%]	1.21 (0.15) [−47%]	0.86 (0.25) [−62%]	<0.0001
Urinary Magnesium (mmol/L)	2.39 (0.24)	0.66 (0.19)	1.00 (0.30) [52%]	1.15 (0.20) [74%]	1.55 (0.24) [135%]	1.72 (0.19) [160%]	<0.0001
Urinary Phosphorus (mmol/L)	0.99 (0.19)	2.37 (0.13)	1.90 (0.22) [−20%]	1.71 (0.12) [−28%]	1.57 (0.18) [−34%]	1.20 (0.22) [−49%]	<0.0001
Urinary Uric Acid (mmol/L)	0.02 (0.007)	0.08 (0.009)	0.05 (0.009) [−38%]	0.05 (0.004) [−38%]	0.04 (0.007) [−50%]	0.04 (0.004) [−50%]	<0.0001
Alkaline Phosphatase (nKat/L)	212.53 (38.88)	1111.39 (41.89)	907.33 (50.21) [−18%]	860.25 (44.03) [−23%]	740.67 (30.19) [−33%]	619.22 (46.90) [−44%]	<0.0001
Serum Urea (mmol/L)	7.68 (0.42)	22.00 (0.80)	18.73 (0.77) [−15%]	16.69 (0.67) [−24%]	13.23 (0.55) [−40%]	10.53 (1.05) [−52%]	<0.0001
Serum Creatinine (mmol/L)	0.03 (0.002)	0.10 (0.02)	0.07 (0.005) [−30%]	0.06 (0.004) [−40%]	0.05 (0.005) [−50%]	0.05 (0.002) [−50%]	<0.0001
Serum Uric Acid (mmol/L)	0.09 (0.01)	0.36 (0.04)	0.30 (0.03) [−17%]	0.27 (0.03) [−25%]	0.19 (0.03) [−47%]	0.16 (0.02) [−56%]	<0.0001
**Lipid Peroxidation and antioxidant estimation**							
Lipid Peroxidation (n moles of MDA/mg Protein)	0.47 (0.08)	2.05 (0.49)	1.64 (0.34) [−20%]	1.03 (0.24) [−50%]	0.90 (0.29) [−56%]	0.70 (0.23) [−66%]	0.0003
Catalase (µ moles of H_2_O_2_/min/mg Protein)	2.85 (0.90)	7.27 (1.02)	5.00 (1.21) [−31%]	3.95 (0.43) [−46%]	3.32 (0.87) [−54%]	3.27 (0.45) [−55%]	0.0004
Reduced Glutathione (n moles of GSH/mg Protein)	14.69 (2.20)	4.66 (0.51)	5.62 (0.47) [21%]	7.26 (1.74) [56%]	10.48 (3.21) [125%]	12.27 (2.34) [163%]	0.0003

The results of the various parameters are shown as Mean (Standard deviation). Percentage difference in body weight between GP 1 and other groups (GP 2–GP 6) are shown in {brackets}. Percentage difference for the various parameters between GP 2 and other groups (GP 3–GP 6) are shown in [brackets]. *The p value for differences of the means among all the 6 groups was calculated using ANOVA. All parameters in GP 2 when compared with parameters in GP 3–6, showed significant difference (p < 0.001), except for urinary calcium and magnesium between GP 2 and GP 3, and urinary magnesium between GP 2 and GP 4. The p values were obtained by Tukey’s test and corrected for multiple testing (please refer to Supplementary Tables 2 & 3 for details).

**Table 3:** Assessment of physiological parameters (body weight, urine biochemistry, serum biochemistry and enzymatic estimation) in rats on curative regimen.

Parameter	GP 1	GP 2	GP 3	GP 4	GP 5	GP 6	P Value*
Body Weight (g)	197.00 (2.83)	112.50 (7.12) {−43%}	130.67 (6.56) {−33%}	143.00 (6.36) {−27%}	155.33 (3.50) {−21%}	162.83 (7.60) {−17%}	<0.0001
Urinary Calcium (mmol/L)	0.30 (0.08)	3.02 (0.45)	2.47 (0.31) [−18%]	2.14 (0.41) [−29%]	1.13 (0.25) [−63%]	0.86 (0.19) [−71%]	<0.0001
Urinary Magnesium (mmol/L)	2.59 (0.36)	0.42 (0.20)	0.80 (0.31) [91%]	0.92 (0.17) [119%]	1.47 (0.25) [250%]	2.01 (0.33) [379%]	<0.0001
Urinary Phosphorus (mmol/L)	0.82 (0.17)	3.55 (0.35)	2.74 (0.26) [−23%]	2.23 (0.36) [−37%]	2.00 (0.19) [−44%]	1.53 (0.26) [−57%]	<0.0001
Urinary Uric Acid (mmol/L)	0.03 (0.02)	0.13 (0.02)	0.06 (0.01) [−54%]	0.06 (0.02) [−54%]	0.05 (0.01) [−62%]	0.04 (0.009) [−69%]	<0.0001
Alkaline Phosphatase (nKat/L)	200.72 (20.9)	1221.89 (39.8)	1013.58 (28.1) [−17%]	965.20 (67.6) [−21%]	771.33 (28.6) [−37%]	730.50 (22.0) [−40%]	<0.0001
Serum Urea (mmol/L)	7.19 (0.49)	23.21 (0.66)	22.00 (0.52) [−5%]	20.12 (0.69) [−13%]	14.51 (0.45) [−38%]	11.9 (0.45) [−49%]	<0.0001
Serum Creatinine (mmol/L)	0.04 (0.003)	0.18 (0.01)	0.16 (0.004) [−11%]	0.12 (0.02) [−33%]	0.09 (0.006) [−50%]	0.06 (0.003) [−67%]	<0.0001
Serum Uric Acid (mmol/L)	0.06 (0.003)	0.46 (0.03)	0.34 (0.01) [−26%]	0.30 (0.01) [−35%]	0.27 (0.02) [−41%]	0.21 (0.009) [−54%]	<0.0001
**Lipid Peroxidation and antioxidant estimation**							
Lipid Peroxidation (n moles of MDA/mg Protein)	0.41 (0.05)	2.48 (0.42)	1.98 (0.22) [−20%]	1.10 (0.14) [−56%]	0.92 (0.10) [−63%]	0.85 (0.26) [−66%]	0.0003
Catalase (µ moles of H_2_O_2_/min/mg Protein)	3.22 (0.81)	7.97 (1.96)	6.96 (0.97) [−13%]	6.37 (1.17) [−20%]	5.11 (0.92) [−36%]	4.63 (0.77) [−42%]	0.0004
Reduced Glutathione (n moles of GSH/mg Protein)	20.18 (2.02)	6.58 (0.64)	5.61 (0.47) [−15%]	10.53 (2.77) [60%]	14.21 (3.23) [115%]	16.08 (2.28) [144%]	0.0003

The results of the various parameters are shown as Mean (Standard deviation). Percentage difference in body weight between GP 1 and other groups (GP 2–GP 6) are shown in {brackets}. Percentage difference for the various parameters between GPP 2 and other groups (GP 3–GP 6) are shown in [brackets]. *The p value for differences of the means among all the 6 groups was calculated using ANOVA. All parameters in GP 2 when compared with parameters in GP 3–6, showed significant difference (p < 0.001), except for urinary calcium and magnesium between GP 2 and GP 3, and urinary magnesium between GP 2 and GP 4. The p values were obtained by Tukey’s test and corrected for multiple testing (please refer to Supplementary Tables 2 & 3 for details).

In the hyperoxaluric group (GP2), levels of malondialdehyde (MDA), which is an indirect index of LPO, were significantly increased along with enhanced catalase activity for both PR (Table 2) and CR rats (Table 3). However, the GSH levels were decreased in hyperoxaluric group (Supplementary Table 3), which could be attributed to the high concentration of free radicals leading to oxidative stress. Estimation of catalase revealed that upon treatment, the enzyme levels were effectively reduced compared to the hyperoxaluric rats and led to reduced oxidative stress, alluding to the preventive and curative property of aqueous extract of *T*. *terrestris* and at the same time contributing as an effective antioxidant agent for the injured kidney tissue.

### Microscopic crystal inhibition evaluation in urine and renal tissue

**Figure 2:**
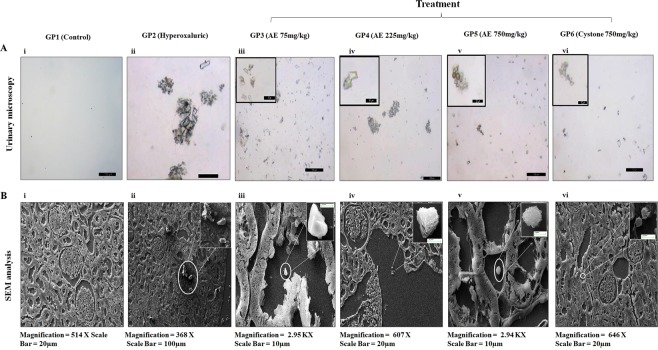
Evaluation of urinary crystals through light microscopy and SEM analysis in rats on prophylactic regimen: (**A**) Microphotographs of excreted urinary crystals, at magnification 10X/20X and (**B**) Scanning electron microscopic architecture and calcium oxalate deposits in renal tissue. GP1: control group, GP2: Hyperoxaluric group, GP3: Calculi induction + treatment of aqueous extract of *T*. *terrestris* at dose 75 mg/kg b.wt., GP4: Calculi induction + treatment of aqueous extract of *T*. *terrestris* at dose 225 mg/kg b.wt., GP5: Calculi induction + treatment of aqueous extract of *T*. *terrestris* at dose 750 mg/kg b.wt. and GP6: Calculi induction + treatment of cystone (positive control) at dose 750 mg/kg b.wt.

**Figure 3:**
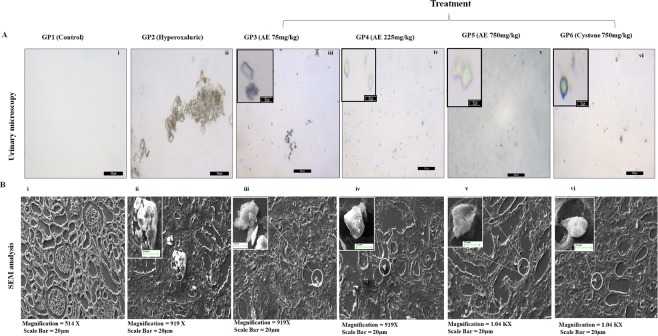
Evaluation of urinary crystals through light microscopy and SEM analysis in rats on curative regimen: (**A**) Microphotographs of excreted urinary crystals, at magnification 10X/20X and (**B**) Scanning electron microscopic architecture and calcium oxalate deposits in renal tissue. GP1: control group, GP2: Hyperoxaluric group, GP3: Calculi induction + treatment of aqueous extract of *T*. *terrestris* at dose 75 mg/kg b.wt., GP4: Calculi induction + treatment of aqueous extract of *T*. *terrestris* at dose 225 mg/kg b.wt., GP5: Calculi induction + treatment of aqueous extract of *T*. *terrestris* at dose 750 mg/kg b.wt. and GP6: Calculi induction + treatment of cystone (positive control) at dose 750 mg/kg b.wt.

Microscopic analysis of urine (Figs 2 and 3) revealed the abundance of large sized COM crystals in the hyperoxaluric rats in PR (Fig. 2A,ii) and in CR (Fig. 3A,ii), whereas the rats treated with the various concentrations of *T*. *terrestris* showed a dose dependent effect on the crystal size and number. Microscopic evaluation of the urine revealed decreased number of crystals which were also seen to be smaller in size and distorted when compared to the hyperoxaluric rats (GP2). Furthermore, scanning electron microscopic analysis (SEM) of kidney sections revealed distortion in crystal morphology, dissolution and reduction in stone size due to treatment. SEM analysis confirmed the preventive (Fig. 2B,iii–v) and curative (Fig. 3Biii–v) efficacy of aqueous extract of *T*. *terrestris* by reducing crystal aggregates, crystal size and also contributing to the morphology modulation as compared to GP2. These effects observed were again comparable to positive control (GP6) group in PR (Fig. 2B,vi) and in curative regimen (Fig. 3B,vi).

### Histopathological findings

**Figure 4:**
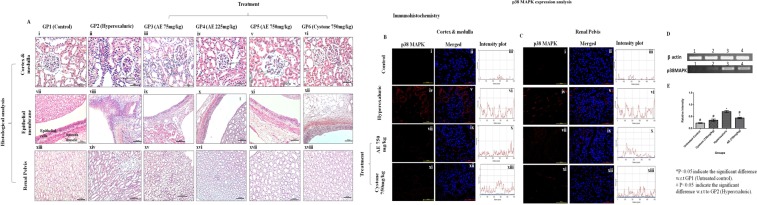
Renal Histology and p38MAPK expression at the gene and protein level in rats on prophylactic regimen: (**A**) Histology of renal tissue of Hematoxylin-Eosin (H&E) stained cortex and medulla, at magnification 20X/40X (**B**) Immunohistochemistry analysis of p38MAPK in cortex and medulla of kidney sections through confocal microscopy, at magnification 60X, (**C**) Immunohistochemistry analysis of p38MAPK in renal pelvis of kidney sections through confocal microscopy at magnification 60X, (**D**) mRNA expression analysis of p38MAPK gene in renal tissue, (**E**) Relative intensity plot of mRNA expression of p38MAPK gene: GP1: control group, GP2: Hyperoxaluric group, GP3: Calculi induction + treatment of aqueous extract of *T*. *terrestris* at dose 75 mg/kg b.wt., GP4: Calculi induction + treatment of aqueous extract of *T*. *terrestris* at dose 225 mg/kg b.wt., GP5: Calculi induction + treatment of aqueous extract of *T*. *terrestris* at dose 750 mg/kg b.wt. and GP6: Calculi induction + treatment of cystone (positive control) at dose 750 mg/kg b.wt.

**Figure 5:**
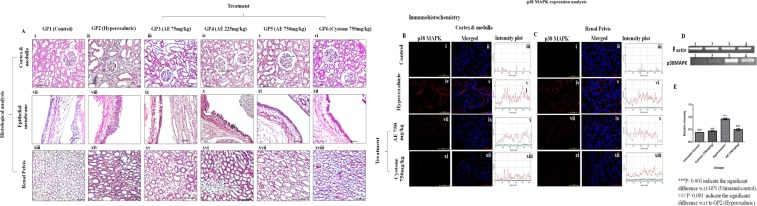
Renal Histology and p38MAPK expression at the gene and protein level in rats on curative regimen: (**A**) Histology of renal tissue of H&E stained cortex and medulla, at magnification 20X/40X, (**B**) Immunohistochemistry analysis of p38MAPK in cortex and medulla of kidney sections through confocal microscopy, at magnification 60X, (**C**) Immunohistochemistry analysis of p38MAPK in renal pelvis of kidney sections through confocal microscopy, at magnification 60X, (**D**) mRNA expression analysis of p38MAPK gene in renal tissue, (**E**) Relative intensity plot of mRNA expression of p38MAPK gene: GP1: control group, GP2: Hyperoxaluric group, GP3: Calculi induction + treatment of aqueous extract of *T*. *terrestris* at dose 75 mg/kg b.wt., GP4: Calculi induction + treatment of aqueous extract of *T*. *terrestris* at dose 225 mg/kg b.wt., GP5: Calculi induction + treatment of aqueous extract of *T*. *terrestris* at dose 750 mg/kg b.wt. and GP6: Calculi induction + treatment of cystone (positive control) at dose 750 mg/kg b.wt.

Histological study confirmed that hyperoxaluric GP2 rats, showed the presence of severe signs of renal injury, including shrinkage in glomerulus and necrosis in cells of cortex and medulla in both PR (Fig. 4A,ii) and in CR (Fig. 5A,ii) rat kidneys. Disruption of smooth muscles of epithelial membrane was also observed in PR (Fig. 4A,viii) as well as CR (Fig. 5A,viii) along with the dilation in ducts of renal pelvis, deposition of hyaline casts in PR (Fig. 4A,xiv) and in CR (Fig. 5C,xiv). On the other hand, treatment with aqueous extract of *T*. *terrestris* significantly reduced these inflammatory signs and led to an overall improvement in the histology of kidney, in dose dependent manner (Fig. 4A) in PR and (Fig. 5A) in CR rats.

### Expression analysis of p38MAPK in renal tissue

To further confirm whether the inflammatory changes seen in the renal tissue had their basis in altered signaling, mRNA expression of p38MAPK from renal tissue was studied through semiquantitative PCR. Due to prominent renal damage, significantly upregulated expression of p38MAPK was observed in hyperoxaluric animals in PR (Fig. 4E) and CR (Fig. 5E) compared to the control group. Treatment at 750 mg/kg b.wt. downregulated the mRNA expression which was evident from the normalized relative intensity graph in PR (Fig. 4E) and CR (Fig. 5E) in comparison to the hyperoxaluric group.

The changes observed at the genetic level were also reflected at the protein level, as documented by the immunohistochemical analysis for p38MAPK, which revealed elevated expression in cortex, medulla and renal pelvis of PR (Fig. 4B,iv–vi,C,iv–vi) and CR (Fig. 5B,iv–vi,C,iv–vi) of hyperoxaluric rats, compared to the weakly positive signals in p38MAPK expression observed in control. The results of immunohistochemical analysis of renal tissue in PR and CR rats treated with *T*. *terrestris* at 750 mg/kg b.wt. revealed decreased p38MAPK expression. These findings have demonstrated that *T*. *terrestris* at 750 mg/kg b.wt., safeguards against inflammatory changes due to elevated p38MAPK expression in renal tissue in response to oxalate injury.

### Preclinical toxicity evaluations

#### Acute oral toxicity evaluation

Clinical assessment was carried out at 10, 30 minutes, 1, 2- and 4-hours following dosing, and daily thereafter during 14-day observation period. No incidence of abnormal clinical signs among the animals was observed. Body weight gain by the treatment was not adversely affected. No gross pathological alterations were observed at necropsy in tissues/organ of any rats. Due to the absence of any adverse effect among treated rats, MTD was estimated to be greater than 2000 mg/kg b.wt.

#### Repeated oral toxicity study

At the end of the treatment period (28 days), oral administration of statistically optimized aqueous extract of *T*. *terrestris* up to the dose of 750 mg/kg b.wt. did not induce any alterations in body weight (Table 4), food consumption or haematological parameters (Table 5). However, slight, non-significant lowering of platelet count at the dose of 225 mg/kg b.wt., mean corpuscular and haemoglobin concentration in male rats at the dose of 225 mg/kg b.wt. and 750 mg/kg b.wt. were incidental because they were comparable to control. Similarly, clinical chemistry parameters (Table 6), few instances differing significantly (p < 0.05) from the vehicle control group values occurred during the study. These included increased alanine aminotransferase (ALT), triglycerides and phosphorus in the female rats at a dose of 225 mg/kg b.wt. and lowering of ALT in male rats treated at 750 mg/kg b.wt. and Aspartate aminotransferase in male rats treated at 75 mg/kg and 750 mg/kg b.wt,but these alterations were considered to be incidental in nature or non-adverse, *i*.*e* no toxicological significance, and the values were within the historical control range of Wistar rats in the test facility. Urinalysis parameters for all dose group animals were found comparable to control at the end of the treatment period. Further, absolute and relative (% body weight) values of liver, kidneys, lungs, adrenals, testes, uterus, brain, spleen and heart did not depict any alterations up-to the dose of 750 mg/kg b.wt. The necropsy examinations did not reveal incidence of any remarkable pathological alterations. Microscopic evaluation of all tissues/organs categorically proved that no incidence of histopathological alterations was seen. The NOAEL of the rats treated with the *T*. *terrestris* was found to be 750 mg/kg b.wt.

**Table 4:** Analysis of body weight changes in Wistar rats during toxicity study.

Treatment period (day)	0	7	14	21	28
**Changes in body weight of male Wistar rats (grams)**					
GP1	193.50 (9.93)	227.67 (12.13)	262.00 (17.12)	290.50 (23.03)	314.33 (28.70)
GP2 (75 mg/kg)	193.17 (11.51)	228.67 (13.47)	263.33 (17.50)	287.67 (24.06)	305.83 (27.97)
GP3 (225 mg/kg)	193.33 (13.95)	226.00 (14.39)	261.00 (12.02)	284.50 (23.18)	310.33 (29.14)
GP4 (750 mg/kg)	194.00 (13.10)	232.17 (13.10)	269.17 (14.88)	296.67 (20.94)	321.83 (26.83)
**Changes in body weight of female Wistar rats** (**grams**)					
GP1	156.33 (8.41)	175.83 (9.62)	188.33 (7.89)	200.50 (6.89)	208.67 (9.40)
GP2 (75 mg/kg)	157.67 (5.57)	176.83 (4.71)	188.50 (6.38)	201.50 (4.32)	209.83 (5.00)
GP3 (225 mg/kg)	156.00 (11.68)	176.17 (10.68)	189.67 (11.69)	201.17 (8.01)	209.83 (6.71)
GP4 (750 mg/kg)	157.50 (5.54)	173.33 (8.87)	188.67 (13.89)	201.83 (14.30)	209.83 (13.75)

The results of the various parameters are shown as Mean (Standard deviation).

**Table 5:** Oral toxicity study: Haematological parameters in Wistar rats.

Groups		GP1	GP2 (75 mg/kg)	GP3 (225 mg/kg)	GP4 (750 mg/kg)		GP1	GP2 (75 mg/kg)	GP3 (225 mg/kg)	GP4 (750 mg/kg)
Parameters										
Hb (g/L)	**Male Wistar rats**	156.8 (5.7)	159.0 (3.9)	160.5 (6.7)	160.2 (4.3)	**Female Wistar rats**	152.0 (5.7)	150.3 (5.0)	151.8(5.7)	152.0 (8.1)
PCV (Proportion of 1.0)		0.44 (0.02)	0.45 (0.01)	0.46 (0.02)	0.46(0.02)		0.44 (0.015)	0.44 (0.015)	0.43 (0.013)	0.44 (0.022)
Total RBC (10^12^/L		8.16 (0.47)	8.39 (0.44)	8.48 (0.51)	8.46 (0.24)		7.89 (0.34)	7.89 (0.34)	8.00 (0.36)	8.00 (0.27)
MCH (fmol/cell)		1.19 (0.03)	1.18 (0.039)	1.18 (0.03)	1.18 (0.03)		1.19(0.07)	1.21 (0.06)	1.18 (0.03)	1.18 (0.03)
MCV (fl)		54.37 (1.25)	54.43 (1.47)	54.55 (1.24)	54.54 (1.53)		55.49 (1.95)	56.09 (2.01)	54.22 (1.28)	54.73 (1.80)
MCHC (g/L)		350.38 (3.6)	351.0 (2.6)	347.4 (4.3)	347.5 (6.0)		347.4 (4.1)	347.4 (8.0)	350.1 (4.5)	347.2 (4.6)
Total WBC (10^9^/L)		5.40 (1.71)	5.45 (1.09)	5.76 (0.99)	6.99 (1.79)		4.92 (1.47)	5.09 (0.61)	5.19 (1.11)	5.78 (1.36)
Neutrophils (Proportion of 1.0)		0.23. (0.06)	0.22 (0.06)	0.24 (0.05)	0.22 (0.05)		0.17 (0.06)	0.16 (0.06)	0.20 (0.07)	0.20 (0.09)
Lymphocytes (Proportion of 1.0)		0.74 (0.07)	0.75 (0.06)	0.73 (0.05)	0.75 (0.06)		0.79 (0.07)	0.80 (0.06)	0.75 (0.07)	0.75 (0.11)
Monocytes (Proportion of 1.0)		0.007 (0.004)	0.005 (0.003)	0.01 (0.01)	0.005 (0.003)		0.006 (0.004)	0.005 (0.002)	0.004 (0.0009)	0.007 (0.004)
Eosinophils (Proportion of 1.0)		0.02 (0.008)	0.03 (0.008)	0.02 (0.007)	0.03 (0.006)		0.04 (0.001)	0.04 (0.002)	0.06 (0.004)	0.04 (0.002)
Basophils (Proportion of 1.0)		0.00004 (0.0001)	0.00007 (0.0002)	0.0008 (0.0001)	0.0009 (0.0001)		0.00004 (0.0001)	0.0003 (0.0004)	0.000 (0.00)	0.0005 (0.0005)
Platelets (10^9^/L)		811.00 (137.79)	800.00 (38.70)	809.67 (82.86)	598.00 (233.68)		961.83 (93.13)	861.67 (289.04)	914.17 (92.69)	1109.67 (113.03)
Reticulocyte Count (Proportion of 1.0)		0.024 (0.004)	0.03 (0.004)	0.003 (0.007)	0.03 (0.01)		0.032 (0.008)	0.028 (0.01)	0.026 (0.009)	0.024 (0.009)
General Blood Picture		NAD	NAD	NAD	NAD		NAD	NAD	NAD	NAD

The results of the various parameters are shown as Mean (Standard deviation).

Hb: Haemoglobin, PCV: Packed cell volume, RBC: Red Blood cells, MCH: Mean corpuscular haemoglobin, MCV: Mean corpuscular volume, MCHC: Mean corpuscular haemoglobin concentration, WBC: White blood cells, NAD: No abnormality detected.

**Table 6:** Oral toxicity study: Electrolytic parameters in Wistar rats.

Groups		GP1	GP2 (75 mg/kg)	GP3 (225 mg/kg)	GP4 (750 mg/kg)		GP1	GP2 (75 mg/kg)	GP3 (225 mg/kg)	GP4 (750 mg/kg)
Parameters										
TP (g/L)	**Male Wistar rats**	65.8 (2.5)	67.3 (4.8)	66.0 (2.3)	67.2 (2.6)	**Female Wistar rats**	67.2 (1.2)	68.2 (4.1)	66.2 (4.4)	66.5 (4.2)
ALB (g/L)		11.7 (1.5)	11.8 (0.8)	11.5 (0.5)	11.7 (0.5)		13.0 (0.9)	12.5 (1.0)	13.0 (1.3)	12.2 (1.3)
ALT (nkat/L)		744.5 (161.8)	605.5 (105.2)	677.8 (105.8)	552.8 (24.5)		469.5 (59)	502.8 (80.5)	666.7 (193.8)	536.2 (57.2)
AST (nkat/L)		1702.8 (204.83)	1436.2 (186.8)	1497.2(131)	1366.7 (123)		1322.2 (227.2)	1330.5 (109.2)	1516.7 (180.1)	1344.5 (164.2)
ALPI (nkat/L)		1941.7 (501.2)	1916.7 (345.83)	1963.8 (510.3)	1972.2 (499.5)		1083.3 (471.5)	1069.5 (410)	1791.7 (821.5)	1747.2 (859.2)
TBI (mmol/L)		0.004 (0.002)	0.005 (0.001)	0.006 (0.001)	0.003 (0.001)		0.002 (0.001)	0.002 (0.0007)	0.002 (0.00)	0.002 (0.00)
Glucose (mmol/L)		5.19 (0.55)	5.61 (0.63)	5.53 (0.43)	5.40 (0.35)		6.02 (0.58)	5.39 (0.41)	5.56 (0.52)	5.65 (0.36)
Cholesterol (mmol/L)		1.74 (0.14)	1.63 (0.24)	1.55 (0.24)	1.76 (0.30)		1.33 (0.1)	1.45(0.21)	1.59 (0.31)	1.56 (0.38)
Blood Urea Nitrogen (mmol/L)		6.67 (1.1)	6.84 (1.3)	6.9 (0.97)	6.9 (2.9)		6.68 (0.95)	7.26 (0.54)	7.32 (0.49)	8.45 (1.42)
Creatinine (mmol/L)		0.039 (0.003)	0.038 (0.004)	0.039 (0.003)	0.042 (0.005)		0.04 (0.08)	0.04 (0.01)	0.04 (0.003)	0.04 (0.004)
TGL (mmol/L)		0.76(0.31)	0.73 (0.34)	0.78 (0.45)	0.59(0.25)		0.34 (0.09)	0.44 (0.08)	0.60 (0.21)	0.53 (0.12)
Sodium (mmol/L)		148.83 (1.17)	148.17 (1.17)	149.50 (0.55)	149.67 (3.08)		147.50 (1.05)	146.67 (1.03)	146.17 (1.83)	147.63 (5.58)
Potassium (mmol/L)		5.47 (0.20)	5.53 (0.15)	5.45(0.30)	5.62 (3.38)		5.15 (0.20)	5.27 (0.50)	5.32 (0.20)	5.38 (0.26)
Calcium (mmol/L)		2.51 (0.06)	2.57 (0.09)	2.55 (0.04)	2.45 (0.05)		2.55 (0.05)	2.59 (0.06)	2.61 (0.03)	2.57 (0.08)
Phosphate (mmol/L)		2.49 (0.08)	2.44 (0.11)	2.53 (0.12)	2.51 (0.24)		2.03 (0.22)	2.11 (0.26)	2.67 (0.6)	2.14 (0.09)
Globulin (g/L)		54.2 (1.9)	55.5 (4.6)	54.5 (2.3)	55.5 (2.5)		54.2 (1.5)	55.7 (4.1)	53.2 (3.9)	54.3 (3.1)

The results of the various parameters are shown as Mean (Standard deviation).

TP: Total protein, ALB: Albumin, ALT: Alanine aminotransferase, AST: Aspartate transaminase, ALPI: Alkaline phosphatase intestinal, TBI: Total bilirubin, TGL: Triglycerides.

## Discussion

In order to optimise the extraction condition for carrying out studies related to plants, we need to define the variables which can impact the extraction conditions, such as temperature, duration, solvent, etc. The choice of the variables that we made were based upon previous studies carried out in our lab using various medicinal plants, wherein we observed that temperature, extraction time and the solid to liquid ratio could impact the outcome of the extraction process^7,10^. The solvent that we chose for our study was water, and the rationale for this was that a majority of medicinal plants used in the Indian traditional system of medicine are first ground into a paste using water, and then consumed orally and therefore we wanted to use a solvent which could directly be translated rather than adopting methods for the removal of volatile extraction solvents.

Various approaches are followed for optimization of extraction conditions, which are categorised as either classical or statistical. In the classical approach, alteration of one parameter at a time (OFAT) is carried out, which is both time consuming and labour intensive and suffers from the inability to effectively discriminate between the cumulative effect amongst the selected variables. Response surface methodology (RSM), is a statistical approach which can take into account every single variable on the overall efficiency of the procedure and has been extensively used to improve the extraction process with a minimal input of experimental data^17^. With the help of RSM the influence of various conditions on extraction procedure can be simulated, both individually and through their cumulative interactions. The results of the RSM data provides dry lab values for optimization of wet lab procedures^18^. In our previous study we had compared the anti-urolithiatic activity of the selected extracts obtained using the RSM approach. The results demonstrated the cytoprotective potential of aqueous extract of *T*. *terrestris* (AE1) in oxalate injured renal epithelial cell lines, in terms of increasing cell viability as well as reducing cell death^7^.

We therefore hypothesized that the selected extract which had effectively shown the ability to modulate crystal morphology and enhance renal cell survival could also be effective in the *in vivo* setting. Therefore, to prove our hypothesis, we used the Wistar rat urolithic model and evaluated the ability of the aqueous extract of *T*. *terrestris* on two separate aspects, namely prevention (prophylactic) and cure (curative) of kidney stones. In order to fulfil these objectives in the prophylactic regimen, we treated the rats with calculi inducing treatment along with various doses of *T*. *terrestris* extract for 15 days. For evaluating whether the extract could possibly attenuate stone formation, the curative regimen was adopted, wherein 15 days of calculi inducing treatment was done in separate group of rats, followed by 13 days of treatment with *T*. *terrestris* extract. It is already well known that the incidence of kidney stones is on the rise and therefore any intervention which could prevent the formation of stones and yet be safe would be highly desirable. A preventive or prophylactic study therefore, would evaluate the ability to inhibit/slow down the process of kidney stone formation in an environment which would otherwise be conducive to stone formation. To evaluate if *T*. *terrestris* extract could be employed as a curative agent, we first induced kidney stone formation as per standardised protocol and then followed it up with the intervention of the extract to mitigate the process of stone formation. As male Wistar rats exhibit a higher rate of stone formation as compared to female rats^19^ therefore, they were selected for this study. Administration of ethylene glycol induces urolithiasis by raising the levels of calcium, uric acid, phosphate and oxalate and facilitates stone deposition in the kidney by disturbing the osmotic balance^20^. Derangement in tubular fluid dynamics can be attributed to intratubular nephrocalcinosis which is the attachment of crystals to dedifferentiated/regenerating epithelial cells of tubules of nephrons and is dependent on both crystal formation and crystal retention^21^.

*T*. *terrestris* is a well-known immunomodulatory, diuretic and antiurolithic agent^6^. Presence of anti-inflammatory, antioxidant biomolecules and diuretic compounds in *T*. *terrestris* extracts were confirmed in our previous study as well as by others^7,22–26^. To validate these results and to see the efficacy and safety of statistically optimized aqueous extract of *T*. *terrestris* for the development of herbal formulation, *in vivo* studies were carried out. To the best of our knowledge this is the first study to establish the preventive and curative antilithic efficacy of statistically optimized aqueous extract of *T*. *terrestris* in experimentally induced nephrolithiasis in male Wistar rats along with its preclinical toxicity for safety evaluation. Our results revealed that the oral administration of aqueous extract of *T*. *terrestris* can potentially maintain the physiological state and metabolic activity of experimental animals by restoring ionic parameters in urine and serum along with an improvement in renal architecture as well as body weight. It is well established that the ionic environment influences the multistep crystallization process and, in this regard, we evaluated key ionic constituents in body fluids which lead to the formation of kidney stones. Elevated levels of calcium in the urine favour the nucleation, aggregation and growth of crystals, ultimately leading to their deposition in the form of kidney stones^25^. Treated animals of both the regimens showed decreased levels of calcium compared to the hyperoxaluric rats. Another component in the body fluid which provides an appropriate environment for deposition of calcium oxalate stone is phosphorous which is present in the form of phosphates^27^. Our findings demonstrated that treatment with aqueous extract of *T*. *terrestris* led to a reduction in the levels of phosphate and was thereby able to reduce the risk of stone formation. The ability of magnesium to inhibit deposition of CaOx stone in renal tubules is due to its property to form a stable complex with oxalate, thereby lowering the concentration of CaOx^28^. A beneficial effect on treatment with the extract was noted as it also led to an elevation of magnesium levels. Uric acid plays a key role in the development of stones as it promotes CaOx deposition by absorbing glutamic acid and other organic compounds^29^. Our findings showed that the concentration of uric acid was reduced after the treatment, which reiterated the antilithic efficacy of aqueous extract of *T*. *terrestris*.

The deposition and binding of CaOx crystals to the apical membranes of renal tubular epithelial leads to membrane damage, which in turn leads to a rise in alkaline phosphatase (ALP) enzyme^30^. Oral administration of aqueous extract of *T*. *terrestris* minimized the renal tubular injury, which was also evidenced by restoration in the levels of ALP in treated groups. It is known that stone deposition arrests the efflux of urine thereby lowering the glomerular filtration rate (GFR). A direct consequence of this is the accumulation of nitrogenous substances (Urea, BUN, creatinine and uric acid) in the blood^31^. However, on treatment with *T*. *terrestris* extract, preventive and curative effects were seen and led to a reduction in accumulated nitrogenous waste in serum and along with increase in the GFR.

Crystal nephropathies have been linked to ROS induced damage and in hyperoxaluric conditions ROS induced renal damage leads to an alteration in the levels of antioxidant enzymes, an increase in LPO, along with impairment in glutathione metabolizing enzyme activity, which can be restored by antioxidant treatment^27,32^. In our previous study, the statistically optimized aqueous extract of *T*. *terrestris* was characterized using FTIR and GC-MS and was shown to contain various bioactive compounds, such as saturated fatty acids, pyrimidines, lanostane terpenoids, oleic acid, Gamabufotalin etc^7,33^. These compounds have been shown to possess antioxidant potential, anti-inflammatory efficacy and also act as potent diuretics. According to scientific data, lanostane terpenoids have been reported to act as reno protectants^34^ as well as anti-inflammatory biomolecules^35^. Therefore, presence of lanostane in our statistically optimized aqueous extract of *T*. *terrestris* could contribute to the renoprotection observed. Deposition of stones lead to the generation of free radicals which cause oxidative damage to proteins, lipids, nucleic acids and cell structure. In order to reduce this damage, it is necessary to maintain the antioxidant activity within the body^36^ and in this regard the presence of antioxidant biomolecules in aqueous extract of *T*. *terrestris* enhances its therapeutic potential^37^. However, when the oxidant stress far outweighs the ability of the antioxidant system to scavenge the free radicals, damage results, as seen in the hyperoxaluric animals, which was manifested by decreased activity of GSH, catalase along with heightened LPO. Treatment with *T*. *terrestris* rebalanced the levels of GSH, LPO and catalase in prophylactic and curative regimens thereby emphasizing the ability of aqueous extract of *T*. *terrestris* to act as an antioxidant formulation. The p38MAPK pathway is activated by various forms of cellular stress and research has shown that exposure of renal epithelial cells to oxalate and calcium oxalate monohydrate (COM)‐crystals leads to its upregulation^38^. In concordance with these findings, we observed elevation in the expression of p38MAPK in hyperoxaluric rats, however the presence of anti-inflammatory compounds in aqueous extract of *T*. *terrestris* significantly downregulated the expression of p38MAPK, which was established through mRNA expression study and immunohistochemistry of kidney sections confirmed by confocal microscopy. Gamabufotalin is a bufadienolides which is present in both animal as well as plant species and has been reported to exert its anti-inflammatory activity by down-regulating the cyclooxygenase (COX)-2 pathway^39^ as well as heat shock proteins^40^. Therefore, our findings shed light on the fact that treatment with *T*. *terrestris* improved the overall animal health not only by balancing the antioxidant level also by regulation of p38MAPK expression. Results of microscopic analysis of urine also lend support to the anticalcifying efficacy of aqueous extract of *T*. *terrestris*. In the urine of the hyperoxaluric GP2 rats, large COM crystals were clearly observed whereas, in the treated groups lesser number of crystals of reduced size were excreted out. Observations from SEM analysis revealed that tissue sections of extract treated groups had distorted, smaller sized crystals and decreased crystal depositions with respect to GP2 in both prophylactic and curative regimens and are consistent with studies which have pointed to the ability of bioactive compounds to modulate crystal size and shape^7,25^. Marked histological changes seen due to CaOx crystal deposition in the hyperoxaluric group were significantly minimized following treatment with the extract. Based on our *in vivo* investigation observations, we can hypothesize that the modulation of crystal shape and number can be attributed to the bioactive compounds of *T*. *terrestris*.This could lead to reduction of the crystal induced membrane damage and the attenuation of inflammatory signaling and also potentially facilitate removal of stones from the body.

Efficacy is not the sole criteria for developing a potential formulation, but in addition evaluation of drug safety which includes investigation of tolerability dose and assessment of side effects are also mandatory and need to be performed according to Good Laboratory Practice standards^41^. Therefore, to assess the safety of the herbal formulation we carried out preclinical testing in rats of both sexes. Our present study has demonstrated that oral administration of extract is safe up-to the dose 2000 mg/kg b.wt. as, no adverse clinical signs, gross pathological changes or mortality were reported in the animals during acute oral toxicity studies. For tolerability evaluation, repeated dose toxicity study was conducted which also revealed the safety of maximum dose *i*.*e* 750 mg/kg b.wt. in male as well as in female rats. Data with regard to the oral bioavailability of aqueous *T*. *terrestris* are however lacking and therefore studies which look into increasing the bioavailability and reducing the effective dose for preventing and curing kidney stones need to be undertaken.

## Conclusions

In conclusion, the present study has demonstrated that the statistically optimized aqueous extract of *T*. *terrestris* has potential prophylactic and curative abilities against experimentally induced nephrolithiasis, acting through various steps, to potentially inhibit the multistep process of stone formation. Findings of acute oral toxicity study establishes the MLD, LD_50_ and MTD of extract to be greater than 2000 mg/kg b.wt. Based on the observations of repeat dose 28 day oral toxicity study, the extract was seen to be safe even at the maximum dose of 750 mg/kg b.wt., as demonstrated by “no observed adverse effect level” (NOAEL). This study throws light on the antiurolithic potential and preclinical efficacy of statistically optimized aqueous extract of *T*. *terrestris*, however strategies which could increase the oral bioavailability of the extract, such as nano-formulations are warranted. This could lead to pilot studies in patients with asymptomatic kidney stones for evaluating safety and efficacy of the extract, in order to provide an effective intervention for not only preventing, but also possibly curing kidney stones.

### Research involving Human Participants and/or Animals

Animals were involved in the research and protocols incorporated were approved by Institutional animal ethics committee and maintained as per the principles and guidelines of the Ethics Committee of Animal Care of Panjab University and in accordance with the Indian National Law and NIH publication No. 86–23(1985), revised in 1996 on animal care and use.

## Supplementary information

Supplementary Information

## Data Availability

All the necessary data will be provided on request.
