# Supplementary material for: Delving into the Antiurolithiatic Potential of Tribulus terrestris Extract Through –In Vivo Efficacy and Preclinical Safety Investigations in Wistar Rats
Source: Sci Rep. 2019 Nov 4;9:15969. doi: 10.1038/s41598-019-52398-w (PMC6828970; doi:10.1038/s41598-019-52398-w)
Supplement: Supplementary file 1 — Supplementary Information [file 41598_2019_52398_MOESM1_ESM.docx]

**Supplementary Data**

**Delving into the Antiurolithiatic Potential of *Tribulus terrestris* Extract Through –*In Vivo* Efficacy and Preclinical Safety Investigations in Wistar Rats**

Jyoti Kaushik^a^, Simran Tandon^b^, Rishi Bhardwaj^c^, Tanzeer Kaur^c^, Surinder Kumar Singla^d^, Jitender Kumar^a^ and Chanderdeep Tandon^a*^

^a^Amity Institute of Biotechnology, Amity University Uttar Pradesh, Noida, India

^b^Amity Institute of Molecular Medicine & Stem Cell Research, Amity University Uttar Pradesh, Noida, India

^c^Department of Biophysics, Panjab University, Chandigarh, India

^d^Department of Biochemistry, Panjab University, Chandigarh, India

***Corresponding author**

Prof. (Dr.) Chanderdeep Tandon

Director,

Amity Institute of Biotechnology,

J-3 Block, Amity University Campus,

Sector – 125,

Noida – 201313 (U.P.)

India

E-mail: ctandon@amity.edu

Phone: (O) 91-120-4392195 (Direct), 09871003672 (Cell)

Fax: 91-120-4392947

**Supplementary Table 1:** Comparison of different groups for urine and serum biochemistry in prophylactic regimen.

| **Groups** | **Body Weight** | **p Value** | **U Ca** | **P Value** | **U Mg** | **P Value** | **U P** | **P Value** | **U UA** | **P Value** | **ALP** | **P Value** | **S U** | **P Value** | **S C** | **P Value** | **S UA** | **P Value** |
| --- | --- | --- | --- | --- | --- | --- | --- | --- | --- | --- | --- | --- | --- | --- | --- | --- | --- | --- |
| 2 vs 1 | -72.50 | 0.0001 | 7.97 | 0.0001 | -4.20 | 0.0001 | 4.28 | 0.0001 | 1.03 | 0.0001 | 53.93 | 0.0001 | 40.08 | 0.0001 | 0.80 | 0.0001 | 4.47 | 0.0001 |
| 3 vs 1 | -50.67 | 0.0001 | 6.46 | 0.0001 | -3.37 | 0.0001 | 2.82 | 0.0001 | 0.42 | 0.0001 | 41.69 | 0.0001 | 30.94 | 0.0001 | 0.38 | 0.0001 | 3.46 | 0.0001 |
| 4 vs 1 | -29.67 | 0.0001 | 5.73 | 0.0001 | -3.00 | 0.0001 | 2.25 | 0.0001 | 0.35 | 0.0001 | 38.86 | 0.0001 | 25.21 | 0.0001 | 0.34 | 0.0001 | 3.01 | 0.0001 |
| 5 vs 1 | -24.83 | 0.0001 | 3.67 | 0.0001 | -2.04 | 0.0001 | 1.79 | 0.0001 | 0.27 | 0.002 | 31.69 | 0.0001 | 15.53 | 0.0001 | 0.22 | 0.03 | 1.72 | 0.0001 |
| 6 vs 1 | -21.00 | 0.0001 | 2.28 | 0.0001 | -1.61 | 0.0001 | 0.66 | 0.35 | 0.20 | 0.04 | 24.40 | 0.0001 | 7.98 | 0.0001 | 0.12 | 0.50 | 1.14 | 0.004 |
| 3 vs 2 | 21.83 | 0.0001 | -1.51 | 0.028 | 0.83 | 0.14 | -1.46 | 0.001 | -0.62 | 0.0001 | -12.24 | 0.0001 | -9.15 | 0.0001 | -0.42 | 0.0001 | -1.01 | 0.01 |
| 4 vs 2 | 42.83 | 0.0001 | -2.25 | 0.0001 | 1.20 | 0.010 | -2.03 | 0.0001 | -0.68 | 0.0001 | -15.07 | 0.0001 | -14.87 | 0.0001 | -0.46 | 0.0001 | -1.46 | 0.0001 |
| 5 vs 2 | 47.67 | 0.0001 | -4.30 | 0.0001 | 2.16 | 0.0001 | -2.49 | 0.0001 | -0.76 | 0.0001 | -22.24 | 0.0001 | -24.56 | 0.0001 | -0.58 | 0.0001 | -2.75 | 0.0001 |
| 6 vs 2 | 51.50 | 0.0001 | -5.69 | 0.0001 | 2.59 | 0.0001 | -3.62 | 0.0001 | -0.83 | 0.0001 | -29.53 | 0.0001 | -32.10 | 0.0001 | -0.68 | 0.0001 | -3.33 | 0.0001 |
| 4 vs 3 | 21.00 | 0.0001 | -0.74 | 0.60 | 0.37 | 0.86 | -0.56 | 0.52 | -0.07 | 0.89 | -2.83 | 0.41 | -5.72 | 0.001 | -0.05 | 0.98 | -0.45 | 0.59 |
| 5 vs 3 | 25.83 | 0.0001 | -2.80 | 0.0001 | 1.34 | 0.003 | -1.03 | 0.04 | -0.15 | 0.20 | -10.00 | 0.0001 | -15.41 | 0.0001 | -0.16 | 0.18 | -1.74 | 0.0001 |
| 6 vs 3 | 29.67 | 0.0001 | -4.18 | 0.0001 | 1.76 | 0.0001 | -2.16 | 0.0001 | -0.22 | 0.02 | -17.29 | 0.0001 | -22.96 | 0.0001 | -0.26 | 0.006 | -2.32 | 0.0001 |
| 5 vs 4 | 4.83 | 0.81 | -2.06 | 0.001 | 0.97 | 0.055 | -0.46 | 0.71 | -0.08 | 0.80 | -7.18 | 0.0001 | -9.69 | 0.0001 | -0.12 | 0.52 | -1.29 | 0.001 |
| 6 vs 4 | 8.67 | 0.25 | -3.45 | 0.0001 | 1.39 | 0.002 | -1.60 | 0.0001 | -0.15 | 0.18 | -14.47 | 0.0001 | -17.23 | 0.0001 | -0.22 | 0.03 | -1.87 | 0.0001 |
| 6 vs 5 | 3.83 | 0.92 | -1.39 | 0.05 | 0.42 | 0.78 | -1.13 | 0.02 | -0.07 | 0.87 | -7.29 | 0.0001 | -7.55 | 0.0001 | -0.10 | 0.66 | -0.58 | 0.32 |

Mean differences between the groups were reported. P values was calculated using Tukey’s test and were corrected for multiple testing.

U Ca- Urinary Calcium, U Mg- Urinary Magnesium, UP- Urinary Phosphorus, U UA- Urinary Uric Acid, ALP- Alkaline Phosphatase, S U- Serum Urea, S C- Serum Creatinine, S UA- Serum Uric Acid.

**Supplementary Table 2:** Comparison of different groups for urine and serum biochemistry in curative regimen.

| **Groups** | **Body Weight** | **P Value** | **U Ca** | **P Value** | **U Mg** | **P Value** | **U P** | **P Value** | **U UA** | **P Value** | **ALP** | **P Value** | **S U** | **P Value** | **S C** | **P Value** | **S UA** | **P Value** |
| --- | --- | --- | --- | --- | --- | --- | --- | --- | --- | --- | --- | --- | --- | --- | --- | --- | --- | --- |
| 2 vs 1 | -84.50 | 0.0001 | 10.87 | 0.0001 | -5.30 | 0.0001 | 8.45 | 0.0001 | 1.65 | 0.0001 | 61.29 | 0.0001 | 44.85 | 0.0001 | 1.65 | 0.0001 | 6.74 | 0.0001 |
| 3 vs 1 | -66.33 | 0.0001 | 8.67 | 0.0001 | -4.36 | 0.0001 | 5.97 | 0.0001 | 0.50 | 0.005 | 48.77 | 0.0001 | 41.46 | 0.0001 | 1.36 | 0.0001 | 4.67 | 0.0001 |
| 4 vs 1 | -54.00 | 0.0001 | 7.41 | 0.0001 | -4.06 | 0.0001 | 4.37 | 0.0001 | 0.42 | 0.03 | 45.87 | 0.0001 | 36.19 | 0.0001 | 0.93 | 0.0001 | 3.99 | 0.0001 |
| 5 vs 1 | -41.67 | 0.0001 | 3.33 | 0.001 | -2.72 | 0.0001 | 3.67 | 0.0001 | 0.35 | 0.09 | 34.24 | 0.0001 | 20.47 | 0.0001 | 0.57 | 0.0001 | 3.56 | 0.0001 |
| 6 vs 1 | -34.17 | 0.0001 | 2.23 | 0.043 | -1.40 | 0.01 | 2.20 | 0.001 | 0.21 | 0.58 | 31.79 | 0.0001 | 13.20 | 0.0001 | 0.25 | 0.003 | 2.49 | 0.0001 |
| 3 vs 2 | 18.17 | 0.0001 | -2.20 | 0.047 | 0.935 | 0.16 | -2.49 | 0.0001 | -1.15 | 0.0001 | -12.52 | 0.0001 | -3.39 | 0.008 | -0.29 | 0.0001 | -2.07 | 0.0001 |
| 4 vs 2 | 30.50 | 0.0001 | -3.47 | 0.001 | 1.24 | 0.03 | -4.08 | 0.0001 | -1.23 | 0.0001 | -15.42 | 0.0001 | -8.66 | 0.0001 | -0.72 | 0.0001 | -2.75 | 0.0001 |
| 5 vs 2 | 42.83 | 0.0001 | -7.54 | 0.0001 | 2.58 | 0.0001 | -4.78 | 0.0001 | -1.30 | 0.0001 | -27.05 | 0.0001 | -24.38 | 0.0001 | -1.08 | 0.0001 | -3.18 | 0.0001 |
| 6 vs 2 | 50.33 | 0.0001 | -8.64 | 0.0001 | 3.90 | 0.0001 | -6.25 | 0.0001 | -1.44 | 0.0001 | -29.50 | 0.0001 | -31.66 | 0.0001 | -1.40 | 0.0001 | -4.26 | 0.0001 |
| 4 vs 3 | 12.33 | 0.01 | -1.26 | 0.51 | 0.31 | 0.96 | -1.60 | 0.032 | -0.08 | 0.99 | -2.90 | 0.27 | -5.27 | 0.0001 | -0.43 | 0.0001 | -0.68 | 0.003 |
| 5 vs 3 | 24.67 | 0.0001 | -5.34 | 0.0001 | 1.64 | 0.002 | -2.30 | 0.001 | -0.15 | 0.83 | -14.54 | 0.0001 | -20.99 | 0.0001 | -0.79 | 0.0001 | -1.11 | 0.0001 |
| 6 vs 3 | 32.17 | 0.0001 | -6.44 | 0.0001 | 2.96 | 0.0001 | -3.76 | 0.0001 | -0.29 | 0.21 | -16.99 | 0.0001 | -28.26 | 0.0001 | -1.11 | 0.0001 | -2.19 | 0.0001 |
| 5 vs 4 | 12.33 | 0.01 | -4.08 | 0.0001 | 1.34 | 0.01 | -0.70 | 0.71 | -0.07 | 0.99 | -11.63 | 0.0001 | -15.72 | 0.0001 | -0.36 | 0.0001 | -0.44 | 0.11 |
| 6 vs 4 | 19.83 | 0.0001 | -5.17 | 0.0001 | 2.66 | 0.0001 | -2.17 | 0.002 | -0.21 | 0.56 | -14.08 | 0.0001 | -22.99 | 0.0001 | -0.69 | 0.0001 | -1.51 | 0.0001 |
| 6 vs 5 | 7.50 | 0.27 | -1.10 | 0.65 | 1.32 | 0.02 | -1.47 | 0.06 | -0.14 | 0.87 | -2.45 | 0.45 | -7.28 | 0.0001 | -0.33 | 0.0001 | -1.07 | 0.0001 |

Mean differences between the groups were reported. P values were calculated using Tukey’s test and were corrected for multiple testing.

U Ca- Urinary Calcium, U Mg- Urinary Magnesium, UP- Urinary Phosphorus, U UA- Urinary Uric Acid, ALP- Alkaline Phosphatase, S U- Serum Urea, S C- Serum Creatinine, S UA- Serum Uric Acid.

**Supplementary Table 3:** Assessment of enzymatic estimation in rats on curative and prophylactic regimen.

|  | **Curative Regimen** | | | | | | **Prophylactic Regimen** | | | | | |
| --- | --- | --- | --- | --- | --- | --- | --- | --- | --- | --- | --- | --- |
| **Groups** | **LPO** | **p Value** | **Catalase** | **p Value** | **GSH** | **p Value** | **LPO** | **p Value** | **Catalase** | **p Value** | **GSH** | **p Value** |
| 2 vs 1 | 1.59 | 0.0001 | 4.42 | 0.0001 | -10.02 | 0.001 | 1.58 | 0.0001 | 4.42 | 0.0001 | -10.02 | 0.001 |
| 3 vs 1 | 1.17 | 0.005 | 2.15 | 0.08 | -9.07 | 0.001 | 1.17 | 0.005 | 2.15 | 0.08 | -9.07 | 0.001 |
| 4 vs 1 | 0.56 | 0.29 | 1.10 | 0.64 | -7.43 | 0.007 | 0.56 | 0.29 | 1.10 | 0.64 | -7.43 | 0.007 |
| 5 vs 1 | 0.44 | 0.53 | 0.47 | 0.98 | -4.21 | 0.18 | 0.44 | 0.53 | 0.47 | 0.98 | -4.21 | 0.18 |
| 6 vs 1 | 0.23 | 0.93 | 0.42 | 0.99 | -2.42 | 0.68 | 0.23 | 0.93 | 0.42 | 0.99 | -2.42 | 0.68 |
| 3 vs 2 | -0.41 | 0.58 | -2.27 | 0.06 | 0.95 | 0.99 | -0.41 | 0.58 | -2.27 | 0.06 | 0.95 | 0.99 |
| 4 vs 2 | -1.03 | 0.014 | -3.32 | 0.005 | 2.59 | 0.62 | -1.03 | 0.014 | -3.32 | 0.005 | 2.59 | 0.62 |
| 5 vs 2 | -1.15 | 0.006 | -3.95 | 0.001 | 5.82 | 0.036 | -1.15 | 0.006 | -3.95 | 0.001 | 5.82 | 0.036 |
| 6 vs 2 | -1.35 | 0.002 | -4.00 | 0.001 | 7.60 | 0.006 | -1.35 | 0.002 | -4.00 | 0.001 | 7.60 | 0.006 |
| 4 vs 3 | -0.61 | 0.21 | -1.06 | 0.67 | 1.64 | 0.91 | -0.61 | 0.21 | -1.06 | 0.67 | 1.64 | 0.91 |
| 5 vs 3 | -0.74 | 0.10 | -1.68 | 0.23 | 4.87 | 0.10 | -0.74 | 0.10 | -1.68 | 0.23 | 4.87 | 0.10 |
| 6 vs 3 | -0.94 | 0.024 | -1.73 | 0.21 | 6.65 | 0.015 | -0.94 | 0.024 | -1.73 | 0.21 | 6.65 | 0.015 |
| 5 vs 4 | -0.12 | 0.99 | -0.63 | 0.94 | 3.23 | 0.41 | -0.12 | 0.99 | -0.63 | 0.94 | 3.23 | 0.41 |
| 6 vs 4 | -0.33 | 0.77 | -0.68 | 0.92 | 5.01 | 0.08 | -0.33 | 0.77 | -0.68 | 0.92 | 5.01 | 0.08 |
| 6 vs 5 | -0.20 | 0.96 | -0.05 | 1.00 | 1.78 | 0.88 | -0.20 | 0.96 | -0.05 | 1.00 | 1.78 | 0.88 |

Mean differences between the groups were reported. P value was calculated using Tukey’s test. P values were corrected for multiple testing.

LPO- Lipid Peroxidase (n moles of MDA/mg Protein), Catalase (µ moles of H_2_O_2_/min/mg Protein), GSH- reduced glutathione (n moles of GSH/mg Protein)
